# Behavioral variant frontotemporal dementia in patients with primary psychiatric disorder: A magnetic resonance imaging study

**DOI:** 10.1002/brb3.2896

**Published:** 2023-03-02

**Authors:** Benedetta Tafuri, Marco Filardi, Maria Elisa Frisullo, Roberto De Blasi, Giovanni Rizzo, Salvatore Nigro, Giancarlo Logroscino

**Affiliations:** ^1^ Department of Translational Biomedicine and Neuroscience (DiBraiN) University of Bari Aldo Moro Bari Italy; ^2^ Center for Neurodegenerative Diseases and the Aging Brain University of Bari Aldo Moro at Pia Fondazione “Card. G. Panico” Tricase Italy; ^3^ Department of Radiology Pia Fondazione Cardinale G. Panico Tricase Lecce Italy; ^4^ IRCCS Istituto delle Scienze Neurologiche di Bologna Bologna Italy; ^5^ Institute of Nanotechnology (NANOTEC) National Research Council Lecce Italy

**Keywords:** behavioral variant frontotemporal dementia, machine learning, MRI, primary psychiatric disorder, support vector machine

## Abstract

**Background:**

The clinical diagnosis of behavioral variant frontotemporal dementia (bvFTD) in patients with a history of primary psychiatric disorder (PPD) is challenging. PPD shows the typical cognitive impairments observed in patients with bvFTD. Therefore, the correct identification of bvFTD onset in patients with a lifetime history of PPD is pivotal for an optimal management.

**Methods:**

Twenty‐nine patients with PPD were included in this study. After clinical and neuropsychological evaluations, 16 patients with PPD were clinically classified as bvFTD (PPD‐bvFTD+), while in 13 cases clinical symptoms were associated with the typical course of the psychiatric disorder itself (PPD‐bvFTD–). Voxel‐ and surface‐based investigations were used to characterize gray matter changes. Volumetric and cortical thickness measures were used to predict the clinical diagnosis at a single‐subject level using a support vector machine (SVM) classification framework. Finally, we compared classification performances of magnetic resonance imaging (MRI) data with automatic visual rating scale of frontal and temporal atrophy.

**Results:**

PPD‐bvFTD+ showed a gray matter decrease in thalamus, hippocampus, temporal pole, lingual, occipital, and superior frontal gyri compared to PPD‐bvFTD– (*p* < .05, family‐wise error‐corrected). SVM classifier showed a discrimination accuracy of 86.2% in differentiating PPD patients with bvFTD from those without bvFTD.

**Conclusions:**

Our study highlights the utility of machine learning applied to structural MRI data to support the clinician in the diagnosis of bvFTD in patients with a history of PPD. Gray matter atrophy in temporal, frontal, and occipital brain regions may represent a useful hallmark for a correct identification of dementia in PPD at a single‐subject level.

## Graphical abstract

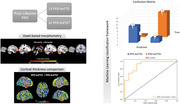

## 1 INTRODUCTION

Frontotemporal lobar degeneration (FTD) is an insidious neurodegenerative disorder and the second most frequent cause of early onset dementia (Logroscino & Piccininni, 2019). Behavioral variant of frontotemporal dementia (bvFTD) represents the most frequent FTD phenotype (Onyike & Diehl‐Schmid, 2013; Ratnavalli et al., 2002), associated with progressive behavioral impairment and early changes in personality (Rascovsky et al., 2011).

In past years, several studies have showed that primary psychiatric disorders (PPD) are often diagnosed in the years preceding a diagnosis of bvFTD (Ducharme et al., 2020; Woolley et al., 2011). While this could be partly due to misdiagnoses (Ducharme et al., 2015), recent investigations have also recognized the possibility that PPD may represent the initial or prodromal stage of bvFTD (Katisko et al., 2019). Indeed, a greater risk of developing dementia has been observed in patients affected by major psychiatric disorders compared to the non‐affected population (Dols et al., 2016; Galimberti et al., 2015; Onyike & Diehl‐Schmid, 2013; Papazacharias et al., 2017). Moreover, epidemiologic investigations have highlighted that half of patients with bvFTD present a history of a previous primary psychiatric disorder before the onset of dementia (Lanata & Miller, 2016; Woolley et al., 2011). Based on these considerations, it is important to take into consideration the bvFTD onset in the disease course of patients with long‐standing psychiatric symptoms. Unfortunately, the identification of frontotemporal dementia in patients with a lifetime mental illness is a diagnostic challenge due to the relatively overlap between bvFTD and PPD in clinical features such as depression, anxiety, obsessive‐compulsive behavior, delusion, euphoria, and personality disorders (Ducharme et al., 2015).

A comprehensive neuropsychological assessment focusing on the domains of attention, memory, executive functions, visuospatial abilities, and language could help differentiate cases (Ducharme et al., 2020); however, patients with bvFTD and PPD share impairments in several cognitive domains (Overbeek et al., 2020). In particular, attention impairments and executive dysfunction represent common features among bvFTD and PPD (Vijverberg et al., 2017). Language impairments can help to distinguish bvFTD from other neurodegenerative disorders but not from specific PPDs (Ziauddeen et al., 2011). In this scenario, a single‐session neuropsychological assessment is often not informative, and longitudinal evaluations, although time and resource demanding, are strongly recommended as the documentation of a progressive cognitive decline is consistent with and underlying neurodegenerative condition (Pressman et al., 2021).

Finally, although frontal and anterior temporal atrophy on magnetic resonance imaging (MRI) has been suggested as a hallmark of bvFTD increasing the diagnostic certainty from “possible” to “probable” (Rascovsky et al., 2011), no studies have evaluated the accuracy of these anatomical changes in characterizing bvFTD in patients with a prior lifetime psychiatric disorder. Indeed, these patients may show gray matter (GM) atrophy in frontotemporal brain regions, making it further difficult to obtain a correct differential diagnosis on an anatomical basis (Ducharme et al., 2020; Selvaraj et al., 2012). Nonetheless, the identification of novel imaging markers able to differentiate between these conditions is of great relevance for patient management and treatment selection.

In the current study, we investigated GM changes in a cohort of patients with a long history of primary psychiatric disorder referred to our center between 2017 and 2019. The study sample consisted of 16 patients with a long history of PPD reclassified as bvFTD (PPD_bvFTD+, among which 10 possible bvFTD and six probable bvFTD) and 13 patients with PPD.

Voxel‐based morphometry (VBM) and cortical thickness were used to explore GM differences between patients with PPD_bvFTD− and PPD_bvFTD+. Moreover, we investigated the usefulness of structural MRI data in supporting the clinical detection of patients with PPD_bvFTD+ at the individual level. To achieve this goal, we evaluated the diagnostic accuracy of a support vector machine (SVM) classification approach in distinguishing PPD_bvFTD+ from PPD_bvFTD− using volumetric and cortical thickness measurements. Finally, we compared classification performances of MRI data to those obtained using automatic visual rating atrophy scale.

## 2 METHODS

### 2.1 Participants

We included 29 patients with a prior long history of primary psychiatric disorder (10 males/19 females; 62.85 ± 6.83 years) and 24 control subjects (8 males/16 females; 63.21 ± 5.91 years).

All participants were referred to the Center for Neurodegenerative Diseases and the Aging Brain in the Department of Clinical Research in Neurology—University of Bari “Aldo Moro” at Foundation “Card.G.Panico” Tricase. After clinical workup, 16 out of 29 PPD patients were classified as bvFTD (PPD_bvFTD+) (Rascovsky et al., 2011). In the remaining 13 cases (PPD_bvFTD−), their symptoms were associated with the natural stage of the psychiatric disorder itself. According to the Diagnostic and Statistical Manual of Mental Disorders, Fifth Edition, we categorized psychiatric disorders considering schizophrenia, bipolar, and psychotic disorders as major PPD, and anxiety and depression as minor PPD. A similar distribution of psychiatric disorders was observed in both PPD_bvFTD− and PPD_bvFTD+ groups. Indeed, there were six patients with a minor psychiatric disorder and seven patients with a major psychiatric disorder in the PPD_bvFTD− group. Eight patients with a minor and a major psychiatric disorder were considered in the PPD_bvFTD+ group. We also included 24 healthy controls. All subjects performed a standardized diagnostic procedure including (a) clinical evaluation and clinical interview with caregivers performed by a neurologist with expertise in dementia and psychiatric disorder, (b) comprehensive neuropsychological assessment, and (c) instrumental examination with MRI 3T and blood chemistry. The neurologist examined patients’ medical history, amnestic data, chart review, and the clinical interview administered to the caregiver and established a new diagnosis of “Possible bvFTD” or confirmed the diagnosis of PPD when the behavioral disturbances were better accounted for a psychiatric disorder (Rascovsky et al., 2011). Thereafter, for all patients with a clinical diagnosis of “Possible bvFTD”, MRI data were used to increase the level of diagnostic certainty with six out of 16 patients showing evidence of frontal and/or anterior temporal atrophy at standard visual atrophy scales and were eventually reclassified as “Probable bvFTD” patients.

The Clinical Dementia Rating Scale (CDR) was administered to evaluate the staging of cognitive decline (Morris, 1993). Mini‐Mental State Examination (MMSE) (Measso et al., 1993) and Frontal Assessment Battery (FAB) (Appollonio et al., 2005) were administered as a global cognitive screening assessment. The control group was selected according to ADNI‐3 criteria (Weiner et al., 2017). None of the controls had a history of neurological or psychiatric illness. All study participants gave written informed consent, and the study was approved by the Institutional Review Board (or Ethics Committee) of ASL Lecce (verbale No. 6, July 25, 2017), according to the Helsinki Declaration.

### 2.2 Neuropsychological assessment

Neuropsychological testing included 11 measures assessing five cognitive domains: memory (Rey Auditory Verbal Learning Test Immediate Recall; Rey Auditory Verbal Learning Test Delayed Recall), executive (Verbal fluency test Digit Span Backward and subtest ECAS—Social Cognition), language (Boston Naming Test, Verbal Fluency test), visuospatial (Clock Drawing Test, Figure Copy test), and attention (Digit Span forward and Trail Making Test A). Cognitive test *z*‐scores were computed and averaged for each domain.

### 2.3 MRI acquisition

Structural images were acquired on a 3T scanner (Philips Ingenia 3.0T) using a Fast‐Field Echo (FFE) T1‐weighted sequence (repetition time = 8.2 ms, echo time = 3.8 ms, flip angle = 8°, resolution = 256 × 256, slices = 200, thickness = 1 mm and field of view = 250 mm). Participants were positioned to lie comfortably in the scanner with various foam pads to ensure head fixation.

### 2.4 Voxel‐based morphometry and cortical thickness analysis

All images were visually inspected for gross structural alterations and artifacts. T1‐weighted images were processed, in parallel on MATLAB (9.7.0), with SPM12 (Statistical Parametric Mapping, Institute of Neurology, London, UK, v7771) and with CAT12 (Structural Brain Mapping Group, Jena University Hospital, Jena, Germany, v1600) using default settings.

The pre‐processing pipeline of CAT12 for VBM analysis included corrections for bias‐field inhomogeneities, segmentation into gray matter, white matter, and cerebrospinal fluid. Diffeomorphic Anatomical Registration Through Exponentiated Lie algebra (DARTEL) algorithm for normalization, and modulation to guarantee that relative volumes were preserved following the spatial normalization procedure. The pre‐processed GM data were smoothed with an 8‐mm full‐width‐half‐maximum (FWHM) isotropic Gaussian kernel. An optimal GM mask was also generated from all smoothed images using the SPM12 Masking toolbox and the Luo–Nichols anti‐mode method of automatic thresholding (Luo & Nichols, 2003; Ridgway et al., 2009). Automated surface‐preprocessing algorithms, implemented in the CAT12 toolbox, were applied for cortical thickness analyses. Estimation of cortical thickness and the central surface was performed in one step, based on the projection‐based thickness method (Dahnke et al., 2013). In this study, topology correction (Yotter, Dahnke, et al., 2011), spherical mapping (Yotter, Thompson, et al., 2011), and spherical registration were carried out. The left and right hemisphere of each participant surface‐based cortical thickness (CT) data were smoothed with a 15 mm FWHM isotropic Gaussian kernel as recommended by the authors of the CAT12 toolbox.

For classification purpose, we used the region‐of‐interest (ROI) analysis tool of CAT12 to extract regional GM volumes and mean CT values in different regions defined by Neuromorphometrics (http://Neuromorphometrics.com) and Desikan‐‐Killiany atlases, respectively (Desikan et al., 2006). Then, volumetric measurements were corrected by age, sex, and estimated total intracranial volume (eTIV). Cortical thickness values were also corrected by age and sex effects.

### 2.5 Visual assessment of frontal and temporal atrophy

A visual assessment of bilateral medial temporal lobe atrophy (MTA) (Scheltens et al., 1992), frontal subscale of Pasquier's Global Cortical Atrophy (GCA‐F) scale (Pasquier et al., 1996), and Koedam's scale of posterior atrophy (PA) (Koedam et al., 2010) was performed using Automatic Visual Ratings of Atrophy (AVRA) (https://github.com/gsmartensson/avra_public), a deep learning‐based toolbox for automatic visual radiological analysis (Mårtensson et al., 2019).

### 2.6 Classification framework

For each subject, we defined a vector of features including adjusted volumetric and cortical thickness measurements. Then, a recursive feature elimination (RFE) method with a leave‐one‐out cross‐validation (LOOCV) splitting was used to define the most informative subset of features. In particular, at each stage of the training estimator, defined by a SVM (Chang & Lin, 2011), all features in the training dataset are ranked by importance, discarding the least important features, prior to rebuilding the model. This process was repeated until the best subset of features was defined. After the selection of the optimal feature subset, we evaluated classification performance with SVM algorithm in a LOOCV splitting of the dataset. During each run, each sample was used once as a test set, while the remaining samples made up the training set. Performance classification was made considering the vector of predictions on test subject at each iteration.

To compare the model with results obtained using visual assessment scales, we have also trained a LOOCV SVM for MTA, GCA‐F, and PA measures and for a combination of them.

### 2.7 Statistical analysis

To check for normality of continuous data, Shapiro‐Wilk test was used. Variables with normal distribution were compared across groups using analysis of variance, followed by pairwise *t*‐tests. Non‐normally distributed variables, instead, were compared across groups using Kruskal–Wallis test, followed by pairwise Mann–Whitney *U* test. The difference in sex distribution among groups was evaluated using Chi‐square test. Differences in neuropsychological functions across groups were assessed using Kruskal–Wallis test, followed by pairwise Mann–Whitney *U* test. Statistical analysis was performed by using R software (Version 3.6.3; R Foundation for Statistical Computing, Vienna, Austria).

In group comparisons, we performed a voxel‐wise two‐samples *t*‐test to study the GM and CT differences between groups. Whole‐brain statistical analyses were performed using the threshold‐free cluster enhancement toolbox (dbm.neuro.uni‐jena.de). This nonparametric permutation‐based approach was performed with 5000 permutations using a significant statistical threshold of family‐wise error rate‐corrected *p* < .05. Differences in GM density between groups were controlled for age, gender, and eTIV. Cortical thickness comparisons were performed defining a general linear model using age and gender as covariates.

Classification performances of SVM were evaluated by accuracy, sensitivity, specificity, and the area under the receiving operating characteristic curve (AUC‐ROC).

## 3 RESULTS

### 3.1 Demographic, clinical, and neuropsychological characteristics of participants

**FIGURE 1:**
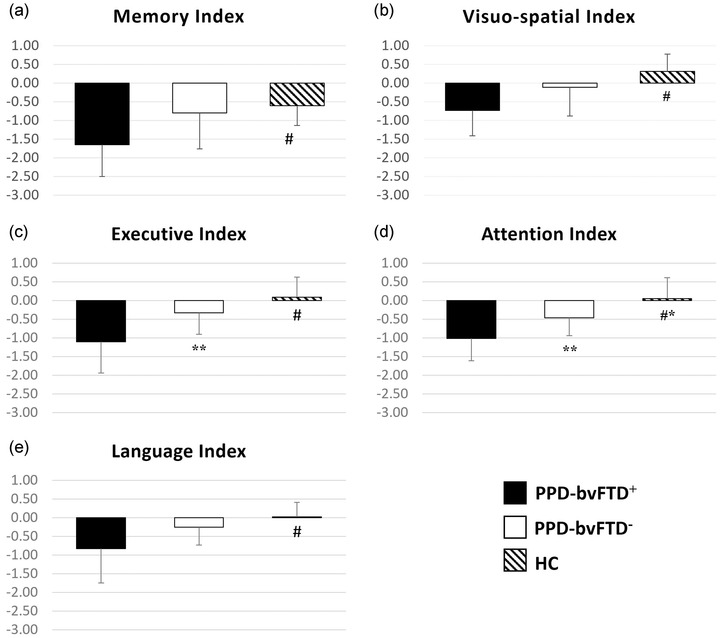
Composite z‐scores for (a) memory, (b) visuo‐spatial, (c) executive, (d) attention, and (e) language functions in patients and healthy controls. #PPD_bvFTD+ < HC. *PPD_bvFTD− < HC. **PPD_bvFTD+ < PPD_bvFTD−. Abbreviations: HC, healthy controls; PPD, primary psychiatric disorders; PPD_bvFTD−, PPD patients without behavioral variant of frontotemporal dementia; PPD_bvFTD+, PPD patients with behavioral variant of frontotemporal dementia.

Demographic and clinical characteristics are reported in Table 1. Sex and age distributions, psychiatric disorder duration, and eTIV were similar across groups. MMSE and FAB scores were significantly lower in the PPD_bvFTD+ and PPD_bvFTD− groups compared to controls (*p* < .001, Bonferroni corrected). The CDR score in PPD_bvFTD+ was significantly higher with respect to PPD_bvFTD− (*p =* .003, Bonferroni corrected). We have also reported mean atrophy scores for each group. Figure 1 summarizes the outcome of the subjects’ neuropsychological assessment battery. PPD_bvFTD+ subjects had significantly worse performance than PPD_bvFTD− in executive and attention scores (*p* < .05; Bonferroni corrected).

**TABLE 1:** Demographic, clinical, and neuroimaging characteristics

Characteristic	HC (*n* = 24)	PPD_bvFTD+ (*n* = 16)	PPD_bvFTD− (*n* = 13)	*p*‐Value
Age (years)	63.21 ± 5.91	65.81 ± 7.51	59.54 ± 5.99	ns
Sex (M/F)	8/16	6/10	4/9	ns
Psychiatric disorder duration (month)	–	230.3 ± 155.1	192.92 ± 132.4	ns
Frontotemporal dementia duration (month)	–	34.5 ± 24.8	–	–
MMSE	28.04 ± 1.65	24.8 ± 2.83	25.8 ± 2.89	<.001^a^
FAB (*z*‐score)	−0.38 ± 1.00	−2.66 ± 2.90	−3.27 ± 2.92	.001^b^
CDR score	–	1.12 ± 0.83	0.35 ± 0.24	.003
eTIV (cm; Onyike & Diehl‐Schmid, 2013)	1334 ± 112	1295 ± 136	1347 ± 118	ns
GCA‐F	0.12 ± 0.24	0.45 ± 0.53	0.05 ± 0.13	.004^c^
MTA left	0.86 ± 0.48	1.31 ± 0.70	0.63 ± 0.35	.004^d^
MTA right	0.77 ± 0.45	1.19 ± 0.62	0.56 ± 0.31	.003^e^
PA	0.36 ± 0.33	0.56 ± 0.57	0.22 ± 0.35	ns

*Note*: Data are presented as mean ± SD unless indicated otherwise. Differences between groups were assessed using analysis of variance.

Abbreviations: CDR, Clinical Dementia Rating; eTIV, estimated total intracranial volume; FAB, Frontal Assessment Battery; GCA‐F, frontal subscale of Pasquier's Global Cortical Atrophy scale; HC, healthy controls; MMSE, Mini‐Mental State Examination; MTA, medial temporal lobe atrophy; PA, Koedam's scale of posterior atrophy; PPD, primary psychiatric disorders; PPD_bvFTD, PPD patients without behavioral variant of frontotemporal dementia; PPD_bvFTD+, PPD patients with behavioral variant of frontotemporal dementia.

^a^ PPD_bvFTD+ < HC, PPD_bvFTD− < HC.

^b^ PPD_bvFTD+ < HC, PPD_bvFTD− < HC.

^c^ PPD_bvFTD+ < HC, PPD_bvFTD− < PPD_bvFTD+.

^d^ PPD_bvFTD− < PPD_bvFTD+.

^e^ PPD_bvFTD− < PPD_bvFTD+.

### 3.2 Voxel‐based morphometry analysis

**FIGURE 2:**
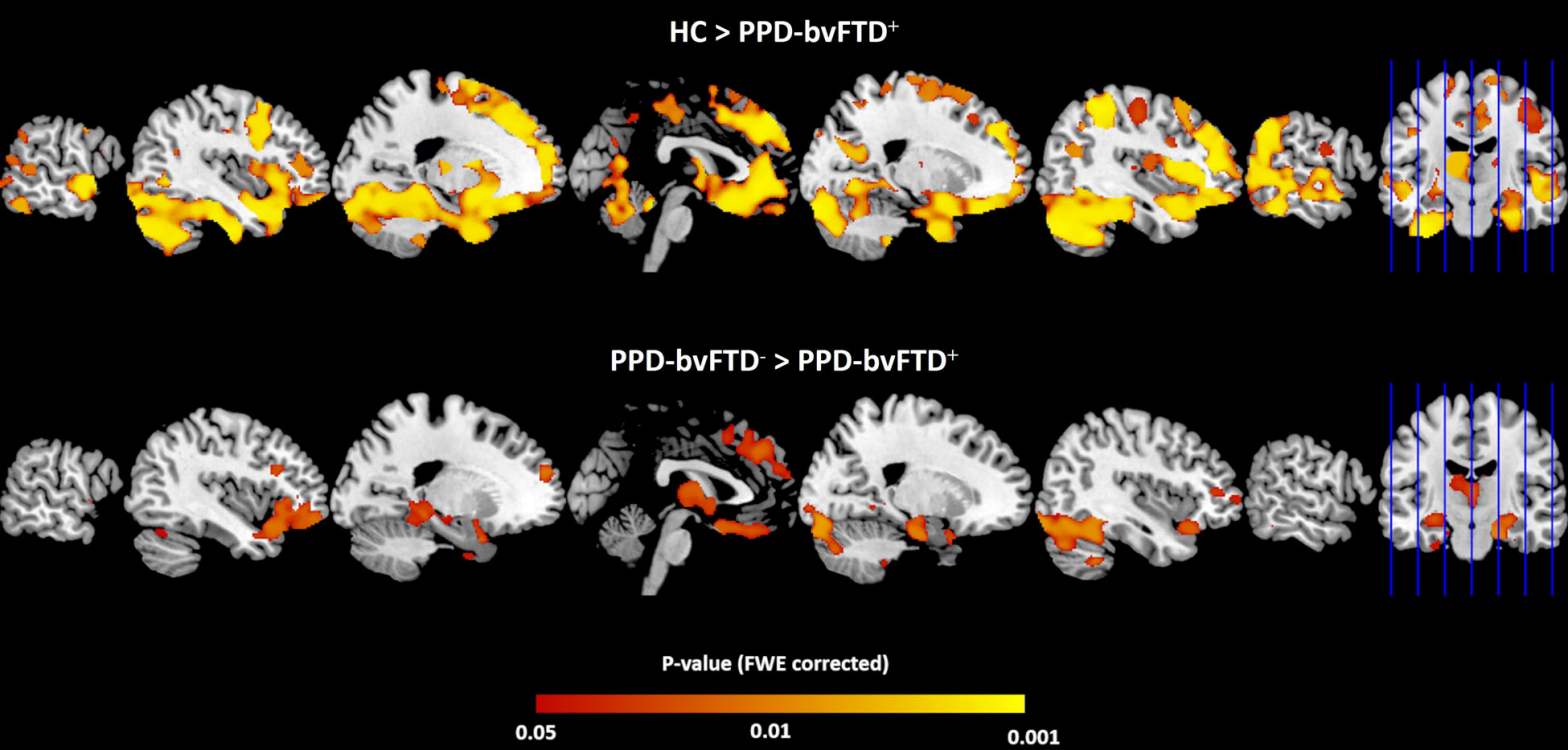
Upper panel: voxel‐wise comparison of GM volume between PPD_bvFTD+ and healthy controls (*p* < .05, FWE‐corrected); bottom panel: voxel‐wise comparison of GM volume between PPD_bvFTD− and PPD_bvFTD+ groups (*p* < .05, FWE‐corrected). Abbreviations: FWE, family‐wise error; GM, gray matter; HC, healthy controls; PPD_bvFTD−, PPD patients without behavioral variant of frontotemporal dementia; PPD_bvFTD+, PPD patients with behavioral variant of frontotemporal dementia.

Compared with controls, patients with PPD_bvFTD+ showed GM atrophy predominantly in the frontal and temporal cortices, cerebellum, bilateral hippocampi, and left thalamus (Figure 2). A similar but less widespread pattern of GM decrease was found between PPD patients with and without dementia. No significant differences were found between PPD_bvFTD− and healthy controls.

### 3.3 Cortical thickness analysis

**FIGURE 3:**
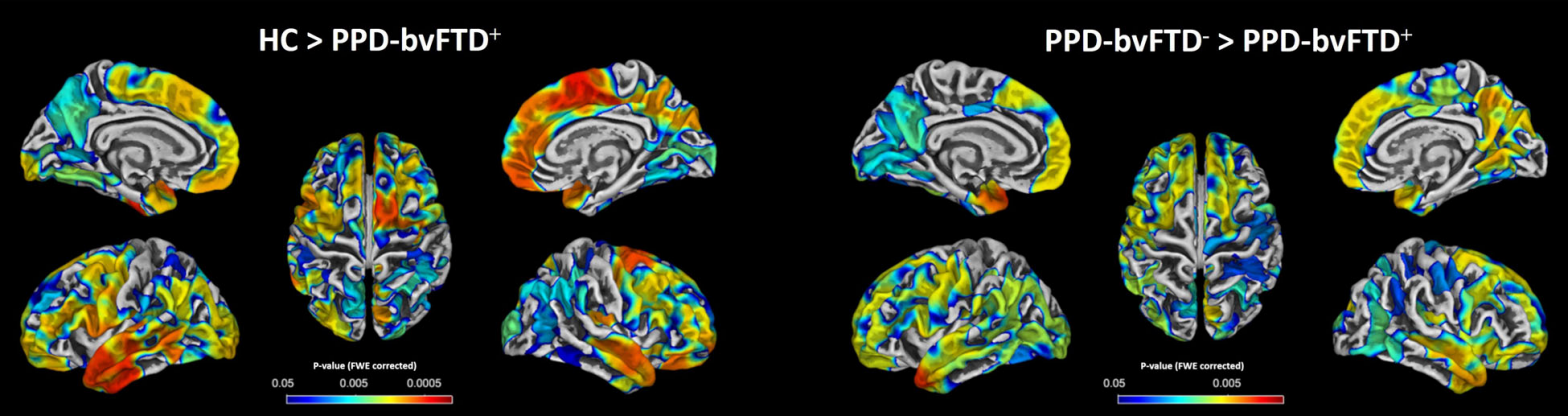
Left panel: vertex‐wise cortical thickness comparison between controls and patients with PPD_bvFTD− (*p* < .05, FWE‐corrected); right panel: vertex‐wise cortical thickness comparison between PPD_bvFTD− and PPD_bvFTD+ (*p* < .05, FWE‐corrected). Abbreviations: FWE, family‐wise error; HC, healthy controls; PPD_bvFTD−, PPD patients without behavioral variant of frontotemporal dementia; PPD_bvFTD+, PPD patients with behavioral variant of frontotemporal dementia.

Relative to patients with PPD_bvFTD− and healthy controls, surface‐based morphometry showed a significant pattern of cortical thinning for patients with PPD_bvFTD+ in frontal, temporal, and occipital lobe bilaterally (Figure 3). In particular, a marked reduction of cortical thickness was observed in the superior frontal gyrus, cuneus, precuneus, superior and middle temporal gyri, and temporal pole. However, no significant differences were found for vertex‐wise cortical thickness analysis between patients with PPD_bvFTD− and healthy controls.

### 3.4 Classification analyses

**FIGURE 4:**
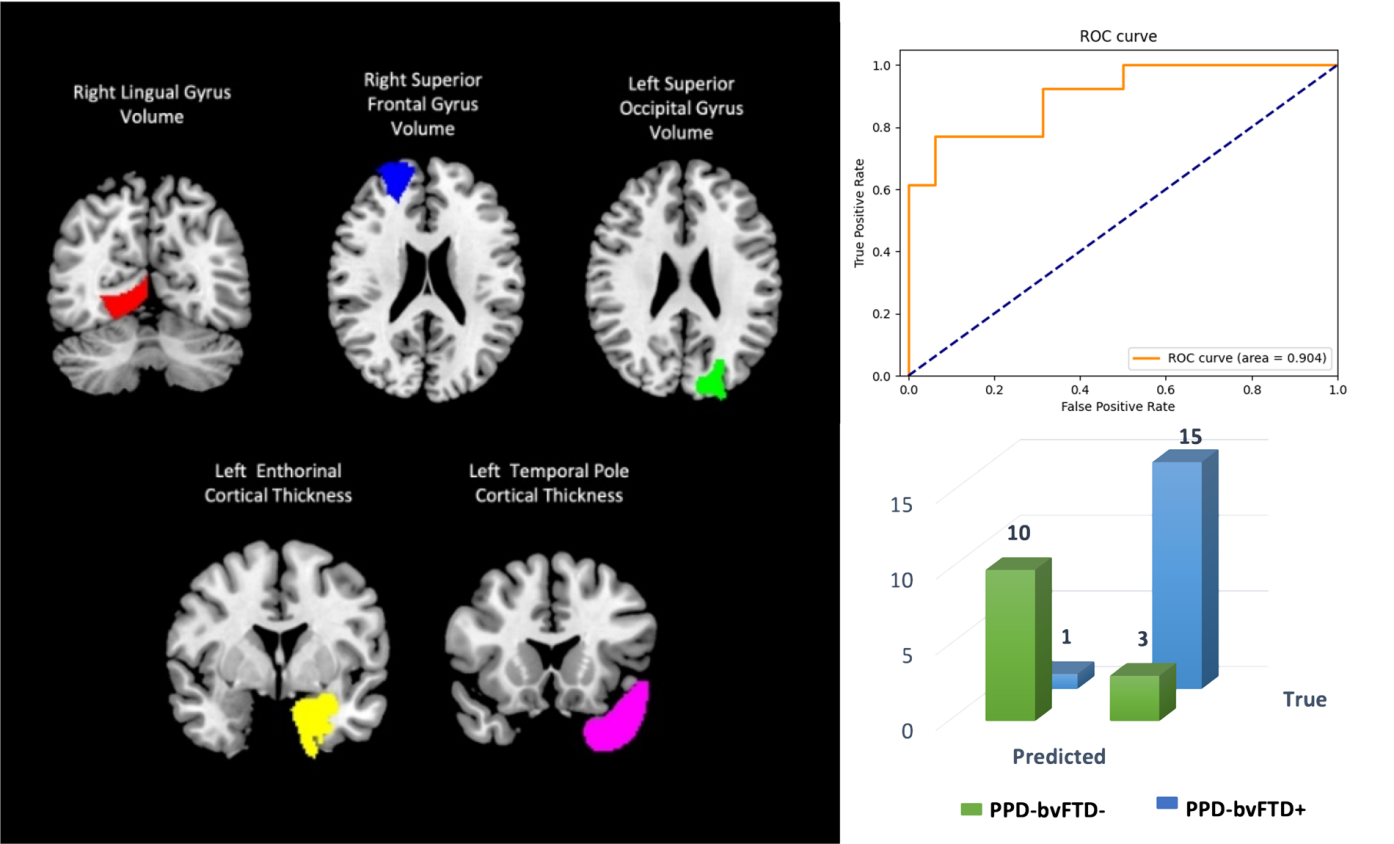
Performance of SVM classification (right panel, ROC curve and confusion matrix) in distinguishing patients with PPD_bvFTD− from those with PPD_bvFTD+, considering the five discriminative features selected by the RFE method (left panel). Abbreviations: AUC, area under the curve; PPD_bvFTD−, PPD patients without behavioral variant of frontotemporal dementia; PPD_bvFTD+, PPD patients with behavioral variant of frontotemporal dementia; ROC curve, receiver operating characteristic curve; SVM, support vector machine; RFE, recursive feature elimination.

RFE feature selection method with LOOCV approach detected a subset of five discriminative features between the PPD_bvFTD− and PPD_bvFTD+ groups. More specifically, cortical thickness of the left entorhinal gyrus and temporal pole and the volumes of the right superior frontal gyrus, left superior occipital gyrus, and right lingual gyrus were discriminatory between these groups. The results of the classification analysis using this subset of features showed an accuracy of 86.2% (sensitivity, 76.9%; specificity, 93.8%) and an AUC of 0.904 (Figure 4). The classification performances for each model are reported in Table 2.

**TABLE 2:** Classification results for trained support vector machine (SVM) models in distinguishing patients with PPD_bvFTD− from those with PPD_bvFTD+

Model	Accuracy (95% CI)	Sensitivity	Specificity	AUC (95% CI)
GCA‐F	0.759 (0.565–0.897)	0.687	0.846	0.841 (0.696–0.986)
MTA	0.759 (0.564–0.897)	0.562	1	0.755 (0.578–0.931)
PA	0.758 (0.565–0.897)	0.625	0.923	0.716 (0.523–0.909)
MTA + GCA + PA	0.793 (0.603–0.920)	0.687	0.923	0.774 (0.603–0.944)
Volume + CT	0.862 (0.683–0.961)	0.769	0.938	0.904 (0.790–1)

Abbreviations: AUC, area under the curve; CT, cortical thickness; GCA‐F, frontal subscale of Pasquier's Global Cortical Atrophy scale; MTA, Medial temporal lobe atrophy; PA, Koedam's scale of posterior atrophy; SVM, support vector machine.

## 4 DISCUSSION

In the current study, we found that patients with PPD_bvFTD+ were characterized by volumetric and cortical thickness reductions in several brain regions such as the thalamus, hippocampus, frontal, temporal cortical, and occipital areas when compared to patients with PPD_bvFTD−. Moreover, morphometric properties showed an optimal accuracy in distinguishing patients at the individual level. Concerning neuropsychological evaluation, patients with PPD_bvFTD+ showed worse neuropsychological functions in executive and attention domains compared to patients with PPD_bvFTD− and healthy controls. Overall, the proposed analytical framework demonstrated a great potential to define a complete morphological description of combined bvFTD and psychiatric illness and help clinician to avoid misclassification of bvFTD with respect to a typical psychiatric subject, caused by symptomatic overlap. Indeed, our findings provided new evidence about morphometric changes associated with bvFTD in patients with a long history of PPD. Different studies have applied machine learning‐based classification of patients with bvFTD based on patterns arising from MRI (Di Benedetto et al., 2022; Meyer et al., 2017; Möller et al., 2016; Tafuri et al., 2022). They have generally reported optimal performance of this approach in distinguishing patients with bvFTD from controls. However, this methodological approach, starting from voxel intensities, overlooked a possible deterioration of cortical surface. On the contrary, we used an ROI‐based approach to take into account neurodegeneration at a surface level, such as cortical thinning, typical of both psychiatric and neurodegenerative conditions, as focus of the studied cohort of patients. In this way, we pointed to combine both volumetric‐ and surface‐based properties in order to construct a more accurate machine‐learning framework. Concerning voxel‐based morphometry analysis, we found GM atrophy in frontal and temporal cortical areas as well as in the hippocampus and thalamus. Cortical changes were also confirmed using surface‐based morphometry showing a marked cortical thinning in fronto‐temporal regions with a strong involvement of the temporal pole in PPD_bvFTD+ when compared to PPD_bvFTD−. These findings were consistent with previous studies describing tissue loss in patients with bvFTD (Du et al., 2007; Nicastro et al., 2020; Whitwell et al., 2015), suggesting that neurodegeneration mechanisms in patients with PPD_bvFTD+ are similar to those observed in the typical presentation of bvFTD. This idea is further supported by subcortical alterations in the hippocampus and thalamus, two subcortical regions strongly involved in FTD pathological processes. Hippocampal changes in the early stage of bvFTD have been widely reported in several postmortem pathology and in vivo imaging studies (Broe et al., 2003; Hornberger et al., 2012; Kril & Halliday, 2004; Seeley, 2008; Whitwell et al., 2009). Specifically, a recent VBM study showed that GM changes in the hippocampus volume were strongly associated with possible visuo‐spatial impairment in patients with bvFTD (Wilson et al., 2020). On the other hand, thalamic atrophy has recently been recognized as a common feature across all FTD phenotype spectra (Bocchetta et al., 2020). Indeed, the thalamus may play a role in the genesis of frontal/behavioral symptoms (Gordon et al., 2016; Pan et al., 2012) associated with emotional, behavioral, and memory dysfunction in patients with bvFTD (Sollberger et al., 2014). Of note, patients with PPD_bvFTD+ showed GM decrease in the cerebellum when compared to patients with typical PPD. Cerebellar damage has been recently associated with cognition and behavioral deficits reported in patients with bvFTD (Schmahmann et al., 2019). Thus, GM alterations observed in our PPD_bvFTD+ group may be linked to the executive function, visuospatial processing, language, and emotion regulation deficits characterizing the onset of bvFTD in patients with a long history of PPD.

As a second aim, we exploited the combination of volumetric and cortical thickness values in defining a classification model. Cortical and subcortical GM changes observed between the PPD_bvFTD+ and PPD_bvFTD− groups allowed to distinguish patients at the individual level. Indeed, the classification analysis based on SVM showed that a specific subset of five features composed by volumetric and thickness values was able to define a discriminative model to differentiate patients with PPD_bvFTD+ and PPD_bvFTD−. In particular, our classification framework achieved an accuracy of 86.2% in distinguishing between patients with PPD_bvFTD− and PPD_bvFTD+ (sensitivity, 76.9%; specificity, 93.8%) capturing significant morphometric changes in the temporal pole and the superior frontal gyrus, two brain regions associated with the key symptoms of bvFTD (Galton et al., 2001; Nicastro et al., 2020). The discriminative pattern obtained in the classification analysis also included the left occipital cortex. Although GM alterations in this brain region are not frequent in patients with FTD a recent functional MRI study has reported connectivity alterations in posterior cortical areas of patients with bvFTD when compared to healthy controls (Chandra et al., 2017). Classification performances obtained using cortical thickness and volumetric values were higher than those obtained using visual atrophy scale. Indeed, although visual atrophy rates and volumetric/thickness values showed a similar specificity in distinguishing between patient groups, the sensitivity of thickness and volumetric measures was higher than that detected using atrophy scale scores. This finding suggests that GM changes in patients with PPD_bvFTD+ go beyond the cortical alterations evaluated using visual scales.

Several limitations need to be recognized when interpreting results. First, the diagnosis of bvFTD and PPD was established on the basis of clinical judgment albeit supported by information on the neuropsychological and functional profile of patients. We decided not to use imaging in cases definition to avoid introducing a methodological error of “diagnostic circularity” (i.e., imaging used both in case definition and as an outcome measure), and we used MRI data solely to increase the level of diagnostic certainty from “Possible” to “Probable” bvFTD. Furthermore, information on the disease progression of a neurodegenerative disease would have increased the degree of diagnostic certainty for both bvFTD and PPD cases, but unfortunately these data were neither collected nor available at the time of the study. Second, the study has a relatively small sample size, which may not be entirely representative of the FTD disease spectrum. Third, none of our patients with PPD_bvFTD+ had a histopathological diagnostic confirmation even if clinical evaluation was performed according to the most recent diagnostic criteria for FTD and was carried out by one of the authors with more than 10 years of experience in dementia diagnosis and care. Fourth, the PPD_bvFTD− and PPD_bvFTD+ groups included patients with different categories of psychiatric disorders. Thus, we were not able to detect specific GM changes associated with a specific psychiatric syndrome due to the heterogeneity in brain alterations and clinical symptoms across different psychiatric categories. Finally, due to the cross‐sectional nature of our study, it is not possible to determine whether cortical and subcortical GM changes found in PPD_bvFTD+ are a primary or secondary phenomenon. In the case these alterations predict the onset of bvFTD, they may represent a structural risk factor, that is, areas of vulnerability in the brain that predispose to the dementia onset in patients with a long history of PPD. Alternatively, the voxel‐ and surface‐based morphometry changes in PPD_bvFTD+ may develop as a result of changes in physiological functioning that are hypothesized to be key mechanisms of bvFTD, that is, areas of brain plasticity resulting from changes in personality and executive dysfunctions. Therefore, further longitudinal studies need to be conducted to evaluate GM changes in patients with PPD from early stages to the possible onset of a bvFTD.

In conclusion, our study provides new evidence on the usefulness of machine learning applied to volumetric and thickness measurements in supporting the clinical diagnosis of dementia in patients with a long history of PPD. In particular, a pattern of GM atrophy involving frontotemporal regions such as the temporal pole and superior frontal gyrus may represent a hallmark for the development of bvFTD in patients with PPD. Finally, our study is a starting point for the definition of new imaging biomarkers capable of differentiating, at the individual level, patients with ambiguous behavioral changes.

## CONFLICT OF INTEREST

Giancarlo Logroscino reports personal fees from Roche and Amplifon, outside of the submitted work. The other authors have no potential conflict to disclose.

### PEER REVIEW

The peer review history for this article is available at https://publons.com/publon/10.1002/brb3.2896.

## Data Availability

The data that support the findings of this study are available on request from the corresponding author. The data are not publicly available due to privacy or ethical restrictions.
